# Phubbing Behavior and its Association With Depression, Anxiety, and Stress in Rehabilitation Students in Iran: A Cross‐Sectional Study

**DOI:** 10.1002/hsr2.70416

**Published:** 2025-03-11

**Authors:** Parvin Dibajnia, Mahdi Azizi, Farnaz Fathollahzadeh, Mehdi Rezaei

**Affiliations:** ^1^ Department of Basic Sciences, School of Rehabilitation Shahid Beheshti University of Medical Sciences Tehran Iran; ^2^ Department of Audiology, Student Research Committee, School of Rehabilitation Shahid Beheshti University of Medical Sciences Tehran Iran; ^3^ Research & Education Network in Audiology & Speech Sciences (RENAS) Tehran Iran; ^4^ Department of Orthotics and Prosthetics, School of Rehabilitation Shahid Beheshti University of Medical Sciences Tehran Iran

**Keywords:** Behavior, Communication, Mental Health, Phubbing

## Abstract

**Background and Aims:**

The study aimed to determine the relationship between phubbing behavior and its association with depression, anxiety, and stress among college students of the School of Rehabilitation at Shahid Beheshti University of Medical Sciences.

**Methods:**

This cross‐sectional survey design used a self‐reported questionnaire that included demographic data, DASS21, and a phubbing scale. The Generic Scale of Phubbing was administered to 320 students from four fields of school of Rehabilitation at Shahid Beheshti University of Medical Sciences. Data analysis was done using the 22nd SPSS software version. The Kolmogorov‐Smirnov, Spearman correlation, Mann‐Whitney and Kruskal‐Wallis tests were used for analysis.

**Results:**

According to the non‐parametric tests, the study revealed a significant association between mental health components and the primary study variables. Specifically, there was a statistically significant positive correlation (*p* < 0.05) between all components of mental health (depression, anxiety, and stress) and phubbing. Furthermore, a significant positive correlation (*p* < 0.05) was identified between all dimensions of phubbing (nomophobia, conflict, self‐isolation, and problem confirmation) and the components of mental health.

**Conclusion:**

The final results indicate that 15% of the total variation in phubbing phubbing can be attributed to the variables of depression and educational background. We suggest that phubbing behavior is linked to lower levels of well‐being and mental health.

## 1 Background and Aims

Technology development has revolutionized communication and information access. However, these advancements have increased reliance on smartphones which resulted in phubbing behavior and consequently will damage interpersonal relationships. From 2016 to 2022, worldwide smartphone users increased from 3.668 to 6.567 billion and are expected to reach 7.690 billion by 2027.

The phenomenon of phubbing (PHU), which refers to the excessive use of digital devices, causing individuals to neglect the people around them, resulting in feelings of being ignored, annoyed, or snubbed [1, 2, 3, 4, 5]. The concept of phubbing has gained increasing attention recently. This term, coined by the Macquarie Dictionary in 2012, is derived from the words “phone” and “snubbing”. This is attributable to the technological advancements and the widespread usage of mobile devices and their impact on face‐to‐face communication and social interactions [5, 6, 7]. Phubbing should not be considered as a simple issue; instead, it is a novel and concerning form of technology addiction that affects various aspects of human psychology and social behavior [8].

Phubbers, people with phubbing behaviors, often experience social isolation, leading to increased reliance on social media for validation and attention [9]. Moreover, a positive correlation exists between phubbing and loneliness [10]. Several studies have explored the relationship between family dynamics and phubbing behavior. One study highlighted that family influences can lead young people to engage in phubbing. Research indicates that parental neglect may contribute to phubbing behaviors in adolescents [11], whereas supportive parenting can act as a protective factor [12]. Additionally, privacy concerns among university students may intensify phubbing behaviors when their personal boundaries are not respected [13].

The academic environment has a significant impact on student behavior, attitudes, actions during their daily activities, and the crucial need for up‐to‐date information or news during their studies, phubbing has become as a growing phenomenon among students. Therefore, understanding the relationship between phubbing and mental health among students is crucial for developing effective prevention strategies.

Research has consistently linked excessive smartphone use to negative mental health outcomes, including anxiety and fear of missing out that arise when students are unable to access the latest information, particularly regarding their academic performance and development. This highlights the addictive nature of smartphone usage, as students feel compelled to constantly check their devices for updates. This behavior is further supported by the fact that students frequently check their phones [14, 15].

Smartphone usage can alter communication and social relationship routines and may cause maladaptive behavior. For instance, excessive smartphone use may reduce eye contact and limit connection and interaction, significantly affecting interpersonal relations [16]. This is all the more important since almost 90% of young users prefer messaging instead of face‐to‐face [17].

The prevalence of phubbing among students is particularly concerning, as it can hinder academic performance and development [18]. Although phubbing can be easily seen in any audience that has access to a smartphone, it is frequently seen among university students [19] and is an issue that should also be emphasized. Because this population, which will soon become decision‐makers and producers in the country they grow up in, consists of individuals with incomplete information and attention problems, which can lead to many unforeseen problems.

Given Iran's unique social and cultural context, where education is highly valued and students often experience significant stress, research on phubbing is especially relevant. This study seeks to explore how phubbing contributes to mental health challenges, providing insights that can guide mental health strategies and educational interventions aimed at fostering healthier student interactions. By investigating the relationships between phubbing behavior, depression, anxiety, and stress among rehabilitation students, this study aims to fill an existing research gap and deepen the understanding of these dynamics. The findings could assist educational institutions in Iran in developing policies and programs to mitigate the negative effects of phubbing, thereby enhancing both mental health and academic performance.

## 2 Methods

### 2.1 Participants

In this cross‐sectional study, we conducted an investigation into the phenomenon of phubbing and its association with depression, anxiety, and stress among a group of 320 students from the School of Rehabilitation at Shahid Beheshti University of Medical Sciences (SBUM). Following approval from the Research and Technology Deputy of SBUM (ethics code: SBMU. RETECH. REC 1401.846), we employed a random sampling technique (convenience sampling) to select participants from all four fields of study within the school, namely audiology, occupational therapy, physiotherapy, and optometry. The inclusion criteria for participation were being a student, regularly using a smartphone, and not having a history of psychiatric disorders. Only those who gave their consent to participate in the study were included in the final sample.

### 2.2 Questionnaires

#### 2.2.1 General Phubbing Scale (GPS)

To evaluate phubbing behavior, Chotpitayasunondh developed a general phubbing scale (Appendix 1) which consists of 15 items and 4 subscales, including nomophobia, interpersonal conflict, personal separation, and phubbing confirmation [20]. This questionnaire has been standardized in Iran by Esfahani et al [21], and has been established to have high internal consistency and validity.

#### 2.2.2 Measurement of Depression, Anxiety, Stress (DASS‐21)

The DASS‐21 is a screening tool used to assess mental health symptoms. It is a self‐report questionnaire used to assess symptoms of depression, anxiety, and stress. In the present study, a short version of questionnaire was employed, consisting of seven items for each subscale (Lovibond & Lovibond, 1995). Participants rated the items on a four‐point Likert scale, ranging from 0 (not applicable to me at all) to 3 (applied to me very much, or most of the time). Total scores on the questionnaire range from 0 to 62, with subscale scores ranging from 0 to 21.

The validity and reliability of the DASS‐21 questionnaire in Iran has been conducted by Asghari Moghaddam et al [22]. Validity assessments, including retesting, yielded values of 0.84 for depression, 0.89 for anxiety, and 0.90 for stress. Furthermore, the questionnaire demonstrated good internal consistency, with Cronbach's alpha values of 0.93, 0.90, and 0.92 for depression, anxiety, and stress, respectively [23]. Overall, the DASS‐21 questionnaire has shown to possess good‐to‐excellent internal consistency, stability, convergent validity, discriminant validity, and a three‐factor structure in the Iranian population [23].

#### 2.2.3 Data Analysis

All the data analysis was done using the 22nd version of the SPSS software. The normality of the distribution of data was examined using the Kolmogorov‐Smirnov test. According to the obtained test results, none of the variables have a normal distribution, therefore, non‐parametric tests were used for analysis. The Spearman correlation test, Mann‐Whitney and Kruskal‐Wallis tests were also used for other analysis.

## 3 Results

### 3.1 Study Characteristic

**Figure 1:**
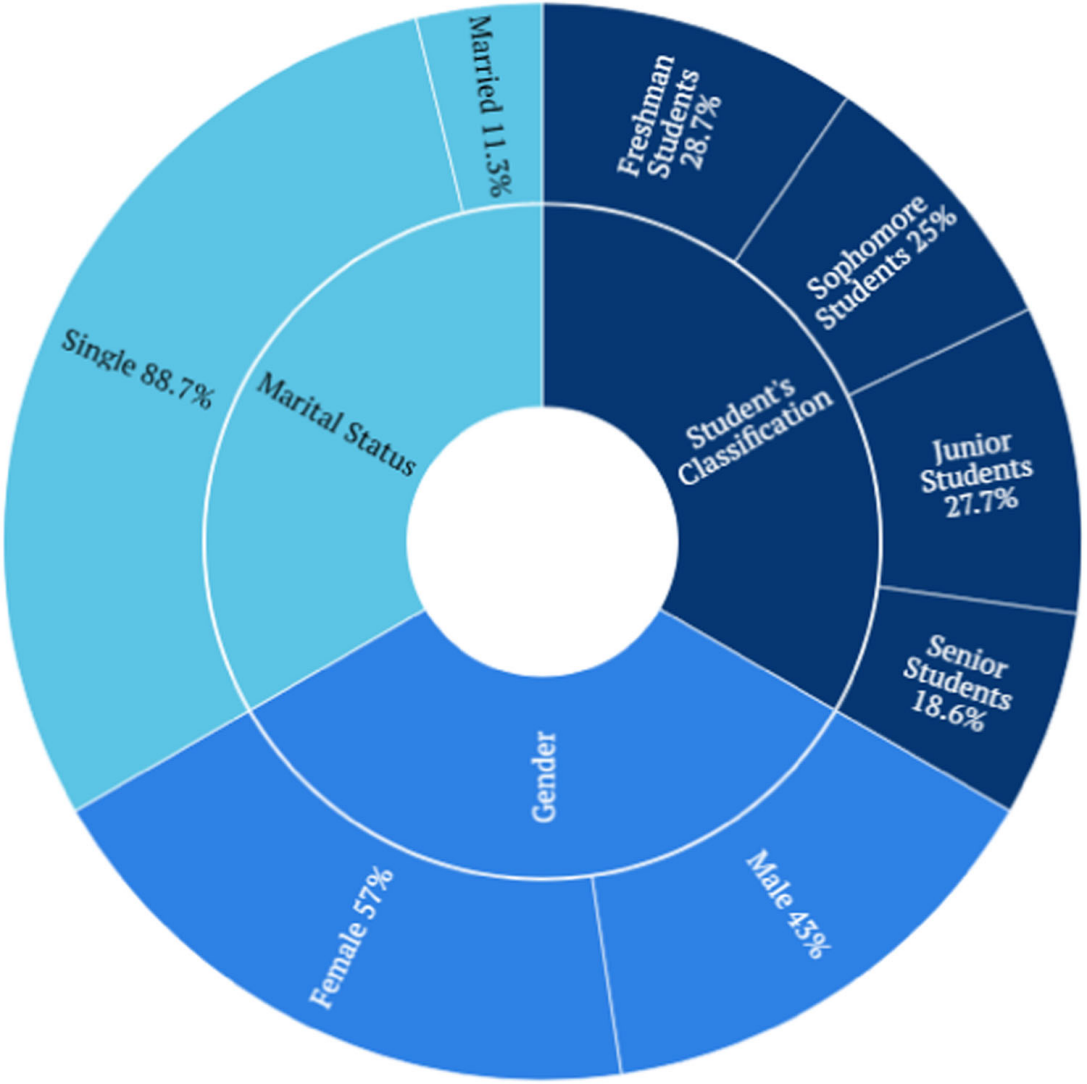
The demographic status of the students.

In our study, we enrolled 320 participants, aged 18 to 42 years (Mage = 21.34 years, SD = 2.63). The majority of participants were Optometry students (*n* = 115, 35.1%), followed by Physiotherapy students (*n* = 82, 25%). Figure 1 illustrates the classification of students, along with their marital status and gender distribution. The participants did not receive any financial incentive for completing the study.

Table 1 presents the main variables measured in this study. The findings indicate that among the participants.

**Table 1:** Mean and standard deviation of the main variables.

Variables	Variation range	Mean	Standard deviation
**Mental health**	0–110	32.3	21.7
Depression	0–38	11.6	7.6
Anxiety	0–38	11.5	8.1
Stress	0–38	9.4	7.8
**Phubbing**	16–72	39.4	9.5
Nomophobia	4–16	12.8	3.8
Conflict	4–16	8.6	3.3
Self‐isolation	3–12	6.2	2.8
Confirmation of the problem	3–12	9.7	2.9

Table 2 investigated psychological and mental health dimensions, including depression, anxiety, and stress.

**Table 2:** Assessment of the status of psychological health dimensions.

**Mental health**	**Percent**	**Frequency**	**Classification**	**Variables**
	**Depression**	Normal	130	39.6
		Mild	86	26.3
		Moderate	73	22.3
		Severe	22	6.7
		Extremely severe	17	5.2
	**Anxiety**	Normal	109	33.2
		Mild	43	13.1
		Moderate	94	28.7
		Severe	31	9.5
		Extremely severe	51	15.5
	**Stress**	Normal	264	80.5
		Mild	21	6.4
		Moderate	28	8.5
		Severe	11	3.4
		Extremely severe	4	1.2
	**Pubbing**	No pubbing	167	50.9
		Slight	149	45.4
		Moderate	12	3.7

### 3.2 Analytical Findings

The normality of the distribution of the data related to the main variables of the study was assessed using the Kolmogorov‐Smirnov test (Table 3).

**Table 3:** Examination of the normality of data distribution for study variables.

Variables	Kolmogorov‐Smirnov test		
	*p* value	Degree of freedom	Statistic
**Mental health**	0.001	328	0.13
Depression			0.14
Anxiety			0.13
Stress			0.14
**Phubbing**			0.07
Nomophobia			0.08
Conflict			0.11
Self‐isolation			0.14
Confirmation of the problem			0.11

As shown in Table 4, none of the variables follow a normal distribution. Consequently, non‐parametric tests were employed in their analysis. The investigation of the relationship between mental health components and the primary study variables reveals a statistically significant correlation.)

**Table 4:** Relationship between study variables and mental health.

Independent variable	Dependent variable	*p*value	Statistic
**Phubbing**	Depression	0.001	0.30
	Anxiety		0.29
	Stress		0.29
**Nomophobia**	Depression		0.23
	Anxiety		0.20
	Stress		0.23
**Conflict**	Depression		0.18
	Anxiety		0.19
	Stress		0.22
**Self‐isolation**	Depression		0.19
	Anxiety		0.25
	Stress		0.19
**Confirmation of the problem**	Depression		0.24
	Anxiety		0.19
	Stress		0.15

The Spearman correlation test was used to investigate the relationship between the dimensions of mental health and the variable of phubbing. The results showed that there is a significant positive correlation (*p* < 0.05) between all components of mental health (depression, anxiety, and stress) and phubbing. Additionally, a significant positive correlation (*p* < 0.05) was found between all dimensions of phubbing (nomophobia, conflict, self‐isolation, and problem confirmation) and the components of mental health.

### 3.3 Multiple Linear Regression (MLR)

Multiple linear regression (MLR) was used in this study to investigate the correlation between the variables being investigated (e.g., anxiety, depression, stress). Following the assumption of normality, the MLR assumption was immediately tested.

In our study, phubbing was considered as the response variable. Other variables that were investigated as independent variables and their relationship with phubbing and their influential mechanism were examined. For this purpose, a multiple linear regression model was performed.

In the summary of the model presented in Table 5, it can be seen that the correlation coefficient (R) between the variables is 0.394, indicating a moderate correlation between the independent variables and the dependent variable of the study. However, the final adjusted determination coefficient, which is equal to 155, shows that 15% of the total variation in phubbing in this study is dependent on the variables of depression and educational background. In other words, independent variables estimate about 13% of the variance of the dependent variable.

**Table 5:** Summary of the model.

R square adjusted coefficient	R square coefficient determination	R correlation coefficient	Model
0.132	0.155	0.394	1

Variables entered the model included: gender, age, marital status, field of study, academic year, depression, anxiety, and stress.

According ANOVA evaluation determines the overall significance of the model. The F value is equal to 6.6 and the *p* value is 0.001, since the level of significance is less than 0.05, it can be concluded that the independent variables explain the variation in the dependent variable well.

In general, the ANOVA indicates that the intended research model is acceptable based on the F statistic value and the probability value that is less than 0.05, and it can explain the variance in the dependent variable. The larger the beta and t values, and the smaller the significant level, the more influence the independent variable has on the dependent variable. In this case, the beta values for educational background (–0.17) and depression (0.26) were not significant, while the other variables entered into the model were significant (Table 6).

**Table 6:** Regression coefficients of independent variables on the dependent variable.

Variable	Standardized coefficients		Unstandardized coefficients	*p* value	*T*
	Beta	Std. error	*B*		
**Constant value variable**		9.78	41.32	0.001	4.18
**Gender**	−0.1	1.05	−2.01	0.58	−1.9
**Age**	0.01	0.26	0.05	0.84	0.2
**Field of study**	−0.17	0.44	−1.38	0.002	−3.13
**Academic year**	0.03	0.58	0.33	0.56	0.57
**Marital status**	−0.01	3.29	−0.98	0.76	−0.29
**Depression**	0.26	0.13	0.33	0.01	2.45
**Anxiety**	0.04	0.12	0.05	0.66	0.43
**Stress**	0.4	0.13	0.05	0.68	0.41

## 4 Discussion

The challenges and risks associated with the increasingly digitalized world are growing, one of which is the phenomenon known as phubbing. Our study aimed to investigate the correlation between phubbing and mental health. While smartphones offer numerous benefits, excessive use has been linked to various psychological issues, including phubbing. In today's interconnected environment, individuals can communicate, learn, and work across distances through digital connectivity. Mobile devices help bridge physical gaps and facilitate social interactions and entertainment; however, excessive smartphone use has been linked to various psychological issues and maladaptive behaviors, including phubbing [24].

It is essential to acknowledge that phubbing is not a trivial concern. Instead, it represents a growing and alarming form of technology addiction that affects both the psychological and social dimensions of people's lives [8].

Our study revealed a significant positive correlation (*p* < 0.05) between phubbing and all components of mental health, including depression, anxiety, and stress. Furthermore, there was a significant positive relationship (*p* < 0.05) between the various dimensions of phubbing—such as nomophobia, conflict, self‐isolation, and problem confirmation—and the components of mental health, indicating that phubbing substantially impacts mental well‐being.

Additionally, our findings are consistent with previous research that identified a positive correlation between phubbing behavior and depression [9, 14, 25]. Ivanova et al. suggested a statistically significant relationship between depressed mood and phubbing behavior [26].

As stated by Wang et al, there exists a “bidirectional” relationship between depression and phubbing behavior. Wang et al. noted a “bidirectional” relationship, wherein depressed individuals may resort to excessive smartphone use to cope with their negative emotions, which can, in turn, exacerbate feelings of depression [27].

Ergun et al. further indicated that individuals experiencing depression tend to engage in phubbing more frequently compared to their nondepressed counterparts, possibly because online communication is perceived as less risky and offers easier access to social support [28]. Furthermore, Sun and Samp stated that phubbing behavior negatively impacts friendship satisfaction, exacerbating depressive symptoms by reducing interpersonal relationships [29]. Some researchers have illustrated that depression, which causes a series of negative perceptions and experiences, such as feelings of inferiority, often leads people to adopt avoidance coping behaviors and to rely on smartphones to cope with their emotional problems or build supportive social relationships [30, 31]. This highlights the complex relationship between depression and smartphone use, where the latter often serves as both a temporary relief and a contributing factor to worsening depression.

For college students, depression can be a significant risk factor for problematic smartphone use [32]. Similarly, individuals with social anxiety are more likely to develop smartphone addiction, as they may prefer online interactions over face‐to‐face communication to avoid distressing situations [33, 34].

Moreover, the emotional fallout from being phubbed—such as negative feelings during social gatherings—can adversely affect mental health [28]. Biologically, the electromagnetic waves emitted by smartphones can disrupt melatonin production, leading to sleep disturbances that correlate with increased depressive symptoms [35, 36].

Individuals may use their phones excessively to cope with underlying psychological disorders, perpetuating a cycle of dysfunction. Depression has become one of the reasons for people to have phubbing behavior [37, 38]. Phubbing also indirectly affects depressive symptoms by diminishing life satisfaction and fostering feelings of social exclusion [4], which can further heighten depressive feelings [1].

Individuals who have lower differentiation of self, are more likely to engage in “phubbing” behaviors through the mediation of fear of missing out (FOMO) [39].

According to the above article, phubbing behavior can act as a defense mechanism, helping these individuals cope with their anxiety about missing out on information. Overall, this highlights a relationship between self‐differentiation, FOMO, and the tendency to engage in phubbing as a coping strategy.

Our results indicated a significant positive correlation (*p* < 0.05) between anxiety and phubbing behavior. This aligns with recent research that suggests individuals with elevated anxiety levels may engage in more phubbing as a means to alleviate discomfort during social interactions [14, 28, 36]. Phubbing is positively correlated with anxiety and has an impact on interpersonal relationships and personal well‐being [1, 40].

By avoiding face‐to‐face contact, students often resort to their smartphones, using them as a refuge from overwhelming feelings [41]. This pattern can impair listening skills [42] and contribute to misunderstandings [43], potentially damaging the future relationships between students and clients in therapeutic settings. The normalization of phubbing among young people can have similarly negative repercussions within educational environments.

Moreover, we observed a significant positive correlation (*p* < 0.05) between stress and phubbing. Previous studies suggest that individuals may experience lower stress levels when communicating online as opposed to in‐person interactions [31].

In a Lebanese context, Bitar et al. validated the Generic Scale of Phubbing and explored the relationships between phubbing and mental health indicators such as depression and anxiety. Their study employed a cross‐sectional design, confirming the scale's validity while examining significant correlations with demographic variables [44].

Meanwhile, Letchumyesswary et al. emphasized the predictive roles of boredom, depression, loneliness, and self‐esteem among Malaysian undergraduates. Their research utilized a cross‐sectional approach, analyzing responses from participants aged 18 to 30. The findings highlighted that boredom, depression, and loneliness positively predict phubbing behavior, while self‐esteem negatively influences it [45]. Collectively, these studies emphasize the detrimental impacts of phubbing on social interactions and mental health, underscoring the need for further research and potential interventions to address this emerging issue in diverse cultural contexts.

People became more prone to use their mobile phones to deal with negative emotions and get rid of stress by communicating with others who may have the same problems to get support and encouragement [46]. Add to this, online communication makes users feel comfortable when expressing themselves and their feeling. These people tend to be constantly engaged with their smartphones to escape negative feelings and manage stress [47].

Notably, our study revealed that 50.9% of respondents did not experience phubbing issues; however, 45.4% reported mild phubbing problems, with an additional 3.7% experiencing moderate issues.

Despite the clear advantages of smartphone usage, emerging research indicates a growing concern regarding its potential adverse effects on mental health and the overall quality of social and academic interactions [27]. A substantial body of research indicates that problematic smartphone use is linked to diminished well‐being and poorer mental health outcomes [48]. These insights underscore the necessity of raising awareness about excessive phone usage and its potential to exacerbate stress and hinder interpersonal relationships. Our findings suggest a pathway for future investigations to focus on interventions that improve the psycho‐educational framework surrounding phubbing behavior.

As we explore the intersection of digital mental health services and phubbing behavior, it becomes clear that while digital platforms offer invaluable tools for extending mental health care, they also present challenges that must be addressed to prevent potential adverse effects. Phubbing has been associated with increased anxiety, depression, and stress. Correlations clearly reflected in our DASS‐21 results. This paradox necessitates a balanced approach that capitalizes on the advantages of eMental Health and electronic mental health and psychosocial support (eMHPSS), as advocated by the recent Lancet protocol paper, while concurrently educating users about responsible digital device use [49].

To effectively integrate eMHPSS, we propose the development of comprehensive guidelines that foster not only mental health access but also digital awareness. Such guidelines should include educational frameworks to enhance digital literacy, promoting healthier interpersonal relationships and reducing the detrimental effects of phubbing. By drawing on the expertise of organizations like WHO and ensuring culturally adaptable frameworks, eMHPSS can be optimized to deliver consistent, ethical, and effective mental health support. Emphasizing these components will help bridge the gap between digital engagement and mental health, transforming potential risk factors into opportunities for improved psychological well‐being [49].

Overall, the implementation of these strategies in our framework aims to ensure that digital mental health services remain a force for good in the face of growing digital engagement, cultivating a more balanced and informed approach to mental health in the digital age. By addressing both the immediate need for accessible mental health interventions and the long‐term impacts of digital habits, our study contributes to a nuanced discourse on modern mental health strategies that support comprehensive well‐being among digital natives.

In conclusion, the presented results shed light on the relationship between phubbing and psychological problems such as depression, anxiety, and stress. It is important to identify the signs of psychological problems and to prepare adequate interventions. University consultation organizations should evaluate students to identify potential symptoms associated with excessive phone use, aiming for early intervention to prevent long‐term complications. Additionally, the increasing smartphone engagement among students correlates with various mental health challenges that impact their quality of life and interpersonal relations.

Despite the contributions of this study, there are notable limitations. The cross‐sectional design restricts the ability to draw definitive cause‐and‐effect conclusions. Future research utilizing longitudinal methodologies may offer deeper insights. Additionally, our sample focused exclusively on university and college students and employed convenience sampling, limiting the generalizability of our findings. Addressing these limitations in future studies will be crucial for advancing our understanding of phubbing and its implications for mental health in broader contexts.

## 5 Conclusion

We found a significant positive correlation (*p* < 0.05) between all components of mental health—depression, anxiety, and stress—and phubbing behavior. Therefore, we recommend that university counseling organizations prioritize the evaluation of students to identify symptoms associated with excessive phone use, including depression, anxiety, and stress. Early intervention is crucial for effective treatment and for preventing potential future complications.

## Limitation

The DASS‐21 provides preliminary insights into mental health symptoms but does not replace comprehensive clinical interviwe and diagnosis. Results are to be used as indicators requiring further validation.

## Suggestions

In contrast to the current cross‐sectional study, a longitudinal investigation could be conducted to more thoroughly assess the relationship between phubbing behavior and mental health, as well as to evaluate changes in phubbing over time. To strengthen the study's findings, we recommend that future research incorporate clinical interview and evaluations alongside DASS‐21 to provide a more in‐depth understanding of mental health symptoms and ensure the robustness of the insights gained. While this study focused on rehabilitation college students, comparative research could also be performed between medical, paramedical, and nonmedical students. Additionally, we propose conducting a study involving patients with different psychiatric disorders to evaluate phubbing behavior in relation to life satisfaction. Furthermore, it is recommended to examine the relationship between family functioning, phubbing behavior, and quality of life among students.

## Author Contributions

**Parvin Dibajnia:** study design and interpretation of result, **Mahdi Azizi:** data collection and preparation of the manuscript, **Farnaz Fathollahzadeh:** preparation of the manuscript and **Mehdi Rezaei:** data collection.

## Ethics Statement

This study obtained its ethical approval from the Ethics Committee of Shahid Beheshti University of Medical Sciences (Code: SBMU. RETECH. REC 1401.846). This study was conducted following all relevant ethical guidelines and regulations to ensure the protection of participants' rights and confidentiality. (Code: SBMU. RETECH. REC.1401.846).

## Conflicts of Interest

The authors declare no conflicts of interest.

## Transparency Statement

Farnaz Fathollahzadeh affirms that this manuscript is an honest, accurate, and transparent account of the study being reported; that no important aspects of the study have been omitted; and that any discrepancies from the study as planned have been explained.

## Data Availability

The data that support the findings of this study are available from the corresponding author upon reasonable request.

## Appendix 1

Table A1

**Table A1:** Mean and standard deviation of phubbing items.

	Items	Mean (SD)
1.	I feel anxious if my phone is not nearby	3.0 (1.2)
2.	I cannot stand leaving my phone alone	3.5 (1.3)
3.	I place my phone where I can see it	3.3 (1.2)
4.	I worry that I will miss something important if I do not check my phone	3.1 (1.2)
5.	I have conflicts with others because I am using my phone	2.2 (1.1)
6.	People tell me that I interact with my phone too much	2.5 (1.2)
7.	I get irritated if others ask me to get off my phone and talk to them	1.9 (1.0)
8.	I use my phone even though I know it irritates others	2.0 (1.0)
9.	I would rather pay attention to my phone than talk to others	2.1 (1.1)
10.	I feel content when I am paying attention to my phone instead of others	2.0 (1.0)
11.	I feel good when I stop focusing on others and pay attention to my phone instead	2.1 (1.0)
12.	I get rid of stress by ignoring others and paying attention to my phone instead	2.2 (1.1)
13.	I pay attention to my phone for longer than I intend to do so	3.1 (1.2)
14.	I know that I must miss opportunities to talk to others because I am using my phone	3.2 (1.2)
15.	I find myself thinking “Just a few more minutes” when I am using my phone	3.3 (1.2)

Abbreviation: SD, Standard deviation.
